# A Data-Driven Approach to Carrier Screening for Common Recessive Diseases

**DOI:** 10.3390/jpm10030140

**Published:** 2020-09-22

**Authors:** Anna V. Kiseleva, Marina V. Klimushina, Evgeniia A. Sotnikova, Mikhail G. Divashuk, Alexandra I. Ershova, Olga P. Skirko, Olga V. Kurilova, Anastasia A. Zharikova, Eleonora Yu. Khlebus, Irina A. Efimova, Maria S. Pokrovskaya, Petr A. Slominsky, Svetlana A. Shalnova, Alexey N. Meshkov, Oxana M. Drapkina

**Affiliations:** 1 National Medical Research Center for Preventive Medicine, Ministry of Healthcare of the Russian Federation, Petroverigsky per., 10, bld. 3, 101000 Moscow, Russia; mklimushina@gmail.com (M.V.K.); sotnikova.evgeniya@gmail.com (E.A.S.); divashuk@gmail.com (M.G.D.); alersh@mail.ru (A.I.E.); ops_70@mail.ru (O.P.S.); olga_kurilova81@mail.ru (O.V.K.); azharikova89@gmail.com (A.A.Z.); elkhlebus@gmail.com (E.Y.K.); BioBank@gnicpm.ru (I.A.E.); MPokrovskaya@gnicpm.ru (M.S.P.); SShalnova@gnicpm.ru (S.A.S.); meshkov@lipidclinic.ru (A.N.M.); drapkina@bk.ru (O.M.D.); 2 Kurchatov Genomics Center-ARRIAB, All-Russia Research Institute of Agricultural Biotechnology, Timiryazevskaya Street, 42, 127550 Moscow, Russia; 3 Faculty of Bioengineering and Bioinformatics, Lomonosov Moscow State University, Leninskie Gory, 1-73, 119991 Moscow, Russia; 4 Institute for Information Transmission Problems, Russian Academy of Sciences, Bol’shoi Karetnyi per., 19, 127051 Moscow, Russia; 5 Institute of Molecular Genetics, Russian Academy of Sciences, Kurchatov Sq., 2, 123182 Moscow, Russia; paslominsky@bk.ru

**Keywords:** cystic fibrosis, *CFTR*, phenylketonuria, *PAH*, alpha-1 antitrypsin deficiency, *SERPINA1*, sensorineural hearing loss, *GJB2*, carrier screening

## Abstract

Genetic screening is an advanced tool for reducing recessive disease burden. Nowadays, it is still unclear as to the number of genes or their variants that are necessary for effective screening. This paper describes the development of a carrier screening custom panel for cystic fibrosis, phenylketonuria, alpha-1 antitrypsin deficiency, and sensorineural hearing loss consisting of 116 variants in the *CFTR*, *PAH*, *SERPINA1,* and *GJB2* genes. The approach is based on the cheapest and fastest method, on using a small number of genes, and on the estimation of the effectiveness of carriers’ detection. The custom panel was tested on a population-based cohort that included 1244 participants. Genotypes were determined by the TaqMan OpenArray Genotyping platform on the QuantStudio 12K Flex Real-Time PCR System. The frequency of heterozygotes in the Russian population was 16.87% or 1:6 (CI95%: 14.76–19.00% by Clopper-Pearson exact method): in *CFTR*—2.81% (1:36), *PAH*—2.33% (1:43), *SERPINA1*—4.90% (1:20), and *GJB2*—6.83% (1:15). The data on allele frequencies were obtained for the first time on a Russian population. The panel allows us to identify the vast majority of carriers of recessive diseases in the population. It is an effective approach to carrier screening for common recessive diseases.

## 1. Introduction

Most recessive diseases are rare; however, together they account for a significant proportion of disease burden [1]. Carrier screening aims at informing parents with a wide array of potential reproductive risks and thus maximizing their reproductive choices in order to reduce the birth of affected offspring. Recently, the expanded carrier screening which includes the screening of many variants in a large number of genes across broad ancestry groups has been developed [2]. However, a number of genes for screening are discussed. According to Guo et al., the screening for 415 genes would identify almost the same quantity of couples at risk of having a child with a severe recessive condition as the screening just for 40 of these genes. A population-specific panel designed to capture 5 to 28 genes with carrier rates >1.0% would identify more than 76% risk of genetic diseases for couples during screening. Therefore, the additional benefit of adding rarer disorders to the panel was very low [3]. Moreover, carrier screening will not identify all individuals who are at risk of the screened conditions. A residual risk of being a carrier always remains [4].

For mass screening, the diagnostic panel should be able to identify most of the carriers, but at the same time be as simple, accurate and cheap as possible. Consequently, we tried in this study to propose a diagnostic panel for carrier screening using the cheapest and fastest method while including a small number of genes and to estimate the effectiveness of carrier detection by assessing the population frequencies of the selected variants identified by this custom panel. We took into account the most common autosomal recessive disorders in Russia that have a well-defined phenotype and a detrimental effect on the quality of life. It was also important that these diseases could be effectively tested by real-time PCR as the cheapest and fastest method for genotyping of the most common variants. Thus, our panel includes the common variants of genes associated with cystic fibrosis (CF), phenylketonuria (PKU), alpha-1 antitrypsin deficiency (A1ATD), and sensorineural hearing loss (SNHL).

CF is caused by alterations in the CF transmembrane conductance regulator (*CFTR*; OMIM #602421) and affects different organ systems, mostly the lungs and pancreas [5]. According to the Cystic Fibrosis Mutation Database, more than 2092 variants of the *CFTR* gene are found [6], and 352 of them are CF-causing [7]. The average frequency of the disease among newborns in Russia is 1:10,250 (0.009%) [8]. CF frequency in various regions of Russia varies from 1:2500 to 1:17,000 (0.04–0.005%) [8]. The creation of the national registry of CF patients [9] allowed obtaining data on the spectrum of *CFTR* variants in Russia.

PKU (OMIM #261600) is a metabolic disorder caused by variants in the phenylalanine hydroxylase gene (*PAH*), resulting in an inability to metabolize the amino acid phenylalanine. PKU is characterized by severe intellectual disability, and may also be accompanied by symptoms such as autism, seizures, motor deficits and behavioral problems [10]. More than 1100 variants of the *PAH* gene are known [11]. The average frequency of the disease among newborns in Russia is 1:7142 [12].

A1ATD (OMIM #613490) is characterized by low serum levels of alpha-1 antitrypsin (A1AT) which may lead to liver disease, early-onset pulmonary emphysema, and rare multi-organ vasculitis [13]. At least 100 allelic variants in the A1AT gene (*SERPINA1*) have been associated with different A1AT plasma levels and functions [13]. The most common genetic reasons for low and aberrant A1AT expression are the PiS allele (rs17580) (expressing 50–60% of A1AT) and the PiZ allele (rs28929474) (expressing 10–20% of A1AT) of the *SERPINA1* gene [14]. The prevalence of A1ATD in Western Europe is estimated at approximately 1:2500 newborns, and, according to the study by Fregonese and Stolk, is dependent on Scandinavian descent [15]. However, the exact prevalence of A1ATD in most populations is unknown, and in many individuals it remains undiagnosed. In Russia, large-scale epidemiological studies on the prevalence of A1ATD have not been conducted. In a random sample of Russians from various regions of the European part of Russia the frequency of the PiZ allele ranged from 0.3 to 1%, while the frequency of the PiS allele ranged from 0.2 to 1.5% [16].

SNHL (OMIM #220290) is one of the most prevalent inherited sensory disorders, affecting about 1:1000 children [17]. Despite the enormous heterogeneity of genetic hearing loss, variants in Gap Junction Beta 2 gene (*GJB2*, OMIM#121011) that encodes connexin 26 (CX26) account for up to 50% of cases of SNHL worldwide [18]. In Russia, the frequency of the heterozygous carriage of *GJB2* variants according to cohort studies is 3.4% [19] and 3.7% [20]. The frequency of SNHL among non-adults in one of the Russian regions is 1:621 [21].

Data on the allele frequencies of variants associated with CF, PKU, SNHL, and A1ATD based on a population study in Russia are not available.

## 2. Materials and Methods

### 2.1. Sampling

Participants for the study were taken from the Epidemiology of Cardiovascular Risk Factors and Diseases in Regions of the Russian Federation Study (ESSE-RF) [22]. The ESSE-RF is a multicenter population-based study, conducted in 2012–2013 in 13 regions of Russia. The multi-stage clustered samples of about 2000 people, aged 25–64, from every region were obtained using Kish methods [23]. Blood samples of all individuals were stored at −70 °C in the biobank of the National Medical Research Center for Therapy and Preventive Medicine.

The sample of this study consisted of participants from ESSE-RF, held in the Vologda region of the North-West Federal District of Russia (ESSE-Vologda). A total of 1244 out of 1642 people from ESSE-Vologda were randomly selected for the study (46% were men), and the average age was 44 ± 12 years. The Vologda region was chosen as a typical region dominated by people of Russian nationality [24].

The study was approved by the Independent Ethic Committee of the National Medical Research Center for Therapy and Preventive Medicine (protocol number 07-03/12 from 03.07.2012) and conducted according to the principles expressed in the Declaration of Helsinki. Informed written consent was obtained from all participants.

DNA was extracted from blood samples using QIAamp^®^ DNA Blood Mini Kit (Qiagen, Hilden, Germany). DNA concentration was measured on NanoDrop OneC Spectrophotometer (Thermo Fisher Scientific, Waltham, MA, USA).

### 2.2. Real-Time PCR

The custom panel for genetic diagnostics using Real-Time PCR was developed on the basis of the TaqMan^®^ OpenArray™ Genotyping platform (Thermo Fisher Scientific, Waltham, MA, USA). This panel enables the evaluation 116 single nucleotide polymorphisms (SNPs), deletions, and insertions of the *CFTR*, *PAH*, *SERPINA1*, *GJB2* genes in 92 DNA samples plus 4 negative controls, simultaneously. The reaction mixture, consisting of a DNA sample in a combination with a 2× TaqMan OpenArray Real-Time PCR Master Mix (Thermo Fisher Scientific, Waltham, MA, USA), was loaded onto OpenArray plates using QuantStudio 12K Flex AccuFill system (Thermo Fisher Scientific, Waltham, MA, USA). The plates were coated with immersion liquid and loaded into the QuantStudio 12K Flex Real-Time PCR System for amplification according to the manufacturer’s standard protocol. Data analysis was performed using the TaqMan Genotyper Software package, version 1.4.0 (Thermo Fisher Scientific, Waltham, MA, USA).

### 2.3. Sanger Verification

Real-time PCR data were validated in selected samples (one to three samples for each genotype of the detected variants) by Sanger sequencing of the PCR products. The nucleotide sequence of PCR products was determined using the ABI PRISM BigDye Terminator v3.1 reagent kit (Thermo Fisher Scientific, Waltham, MA, USA) followed by the analysis of the reaction products on DNA sequencer Applied Biosystem 3500 DNA Analyzer (Thermo Fisher Scientific, Waltham, MA, USA) according to manufacturer’s protocol.

### 2.4. Statistical Analysis

We included a sufficient number of subjects to calculate the carrier frequencies for each of the 4 diseases that we investigated. The carrier frequency was defined as the number of heterozygotes of the studied population on the total number of individuals in the same population, which is approximately twice the allele frequency. Estimated total carrier frequencies were deduced from the genotype frequencies. All statistical analyses were performed using SPSS statistical software [25]. Confidence interval (CI) 95% was estimated by the Clopper–Pearson exact method [26]. The disease frequency (DF, *q*^2^) was calculated using the Hardy–Weinberg equation based on the carrier frequency (2*pq*).

## 3. Results

### 3.1. Development of Custom Panel Including Variants in the CFTR, PAH, SERPINA1, and GJB2 Genes

For the creation of a custom panel, we used data on the frequencies of heterozygous carriage of variants in the *CFTR*, *PAH*, *SERPINA1*, and *GJB2* genes among the population of the Russian Federation and other Caucasian populations, as well as data on frequencies obtained from Russian patient registers and identified in groups of patients in various regions of Russia. The included variants had the highest frequencies across Russian patients and Russian cohorts to date. Total of 116 variants were included in the custom panel: *CFTR* (66 variants), *PAH* (23 variants), *SERPINA1* (10 variants), and *GJB2* (17 variants) (Table S1).

The average accuracy of genotyping—the call rate—using the QuantStudio 12K Flex Real-Time PCR system was 94.2%. The reproducibility of the genotyping results was evaluated on two OpenArray plates on different days by different researchers. As a result, the call rate on one plate was 93%, and on the second was 99%. The reproducibility of the results by parallels was 90%.

For validation of the results, we used DNA sequencing by Sanger. Sanger sequencing was performed on some of heterozygous samples identified using the custom panel, as well as the wild-type homozygous samples as controls. The proportion of confirmed results was 71%. Although the genotype analysis with five assays (C__64676246_10 for genotyping rs74767530, C___656878C_30 for rs77932196, C__34696726_10 for rs104894413, C____594705_10 for rs55819880, and C__26083724_20 for rs78194216) detected some heterozygous samples and one mutant homozygous sample, they were not verified by Sanger, possibly due to the absence of heterozygous samples in the study as positive controls. The proportion of confirmed results by Sanger without them was 90%.

This custom panel for detecting the carriage of common autosomal-recessive diseases was also tested by us earlier on 22 DNA samples of CF patients and showed its effectiveness for diagnosis. CF was identified in 79.5% of cases (with fully established CF-mutant genotypes—68.2%, at least one mutant allele was identified in 90.9% of the cases) [27].

### 3.2. Testing of the Custom Panel for Heterozygous Carriage of Variants in the CFTR, PAH, SERPINA1, and GJB2 Genes in a Russian Population

The results of testing the custom panel are presented in Table 1.

We found 209 allelic variants in 4 genes related to the development of the above-mentioned diseases: *CFTR*—2.81% (1:36), *PAH*—2.33% (1:43), *SERPINA1*—4.9% (1:20), and *GJB2*—6.83% (1:15). Only 17% of all variants included in the panel were detected. Four people were carriers of variants in two genes at once. One sample was homozygous for *GJB2* variant rs35887622. Therefore, the total estimated carrier frequency was 16.87% or 1:6 (CI95%: 14.76–19.00% by the Clopper–Pearson exact method). One variant, rs121912714 (C__64676988_10), in the *SERPINA1* gene was done only in 642 samples due to a technical issue, and it could not be included in the redesign of the panel because this assay was discontinued by the company.

### 3.3. Estimated Burden of Four Common Recessive Diseases in Russia

The HF (frequency of heterozygotes) of variants associated with CF and detected in our study is 2.81% (1:36); consequently, expected DF is 0.019%. According to our data, in the Vologda region, on average, for every 36 people, there is 1 healthy carrier of the nucleotide sequence variant associated with the development of CF. This means that approximately 1 couple in 1296 consists of two carriers of heterozygous variants, respectively, meaning that the risk of having a child who is a patient with CF is 1:5184.

The HF of *PAH* variants is 2.33% (1:43), DF is 0.014%, or in the population of the Vologda region, 1 couple in 1849 consists of two carriers of heterozygous variants and the risk of having a child with PKU is 1:7396.

The total HF of *SERPINA1* variants is 4.9%, or 1:20, DF is 0.06%, or in the population of the Vologda region, 1 couple in 400 consists of two carriers of *SERPINA1* variants and the risk of having a child with A1ATD is 1:1600.

The HF of *GJB2* variants was 6.83% or 1:15, DF is 0.12%, or in the population of the Vologda region 1 couple in 225 consists of two carriers of *GJB2* variants, and the risk of having a child with SNHL is 1:900.

## 4. Discussion

We developed the custom panel for the detection of common autosomal-recessive diseases carriage among Caucasians in Russia, applying a high-throughput genotyping technology on the basis of TaqMan OpenArray Genotyping platform (Thermo Fisher Scientific, Waltham, MA, USA), which is well-proven for the determination of SNPs and large-scale screening [30,31]. This platform is a useful, relatively cheap and reliable tool for application in molecular diagnostic and screening. Average accuracy of genotyping—the call rate using the QuantStudio 12K Flex Real-Time PCR system was 94.2%. The reproducibility of the results by parallels was 90%. Validation of the results using Sanger sequencing also showed the reliability of this platform.

However, one of the significant disadvantages associated with using TaqMan OpenArray Genotyping platform is the lack of a positive control, because the analysis is performed at the same time for a large number of variants in an array. It is theoretically possible to create a pool of DNA consisting of positive control samples, including the most common variants; however, this will require optimization to achieve ideal conditions and will increase the cost of analysis.

Our panel allows us to detect the carriers of variants associated with four recessive diseases: CF, PKU, A1ATD, and SNHL. These diseases are more common than other recessive disorders in Russia, with significant debilitating effects on the quality of life. Given the limitations of real-time PCR in diagnosing spinal muscular atrophy, the causative genes of this disorder could not be added to the panel.

Using our panel, we estimated the allele frequency (AF) of *CFTR*, *PAH*, *SERPINA1*, and *GJB2* variants in the Russian population. This is the first time that the AF of these variants has been estimated on randomly selected individuals of the Russian population. Among 1244 participants from the population-based cohort study ESSE-Vologda, the *CFTR*, *PAH*, *SERPINA1*, and *GJB2* variants were found in 208 individuals. Only 20 out of all 116 variants included in the panel were identified.

*CFTR*. A total of 35 heterozygous *CFTR* variants were found, the most prevalent of them were: rs113993960 (p.F508del) HF—1.69%, which was 60% among all detected *CFTR* variants), rs397508679 (p.L138dup) (HF—0.32% and 11.4% among all detected *CFTR* variants), CFTRdele2.3 (c.54-5940_273+10250del21080) (HF—0.24% and 8.6% among all detected *CFTR* variants), rs121908769 (p.L88Ifs*22) (HF—0.24% and 8.6% among all detected *CFTR* variants). Our results can be compared to the results of other population studies: in the Italian population, overall HF was 1:31 (3.23%) [32], among Caucasian participants, it was 1:29 [33], and in Australia, it was 2.91% [34].

In our study, as in many other population studies, p.F508del (rs113993960) was the most prevalent. This variant is a three-base deletion (c.1521_1523delCTT) that removes a phenylalanine residue at position 508 [35]. This variant is also the most common among CF-causing variants in the European population [36,37]. According to the data of the Russian CF patients register of 2017, the AF of this variant was 52.81% [9]. In a representative sample of residents from the city of Novosibirsk, the HF of p.F508del was 1.15% [38]. In other studies in Russian populations, the HF was 1.5% [39] and 2.25% [40]. Among different world population studies, similar results can be found: in the Italian population 42.6% of all detected CF carriers had p.F508del [32], in the United States using panels with 32 and 69 variants—68.69% and 60.49%, respectively [37].

*PAH*. Twenty-nine heterozygous *PAH* variants were found, the most prevalent of them were p.R408W (rs5030858), p.A403V (rs5030857), p.R261Q (rs5030849), and p.I306V (rs62642934). The most common among the identified *PAH* variants was p.R408W (HF was 1.53%, which was 65.5% among all detected *PAH* variants). This result is consistent with the data obtained in other European populations suggesting that p.R408W is the most common cause of PKU [10,41]. This variant is a missense variant in exon 12 leading to the replacement of arginine with tryptophan in codon 408. Earlier p.R408W was found in 14 studied populations of Eurasia with the highest average HF of 1.3% in the Volga-Ural region [42]. In the study of 400 healthy residents of St. Petersburg, it was shown that HF of p.R408W was 0.75% [40], and in a study of 1000 Russian blood donors, the HF of this variant was 2.4% [39]. 

*SERPINA1*. In our study, three different heterozygous variants of the *SERPINA1* gene were identified, and the two most prevalent were p.E288V (rs17580) (HF was 2.65%, which was 55% among all detected *SERPINA1* variants) and p.E366K (rs28929474) (HF was 2.1% and 43.3% among all detected *SERPINA1* variants).

In clinical practice the greatest importance has the PiZ allele (p.Glu366Lys, rs28929474), which causes the development of A1ATD associated with the accumulation of A1AT protein in hepatocytes and/or incomplete inhibition of neutrophil elastase. The PiZ allele is known to occur in a homozygous state in 95% of Caucasian patients with A1ATD. The PiS allele (p.Glu288Val, rs17580) is also considered pathogenic and responsible for intracellular degradation. The AF of PiS allele frequency is from 2% in northern Europe and up to 8.6% in southern Europe, for PiZ allele is 2% and 1.7%, respectively [43]. According to the recent data, even heterozygous carriage of the PiZ variant increases the risk of liver cirrhosis and is the most powerful risk factor for developing cirrhosis today with non-alcoholic fatty liver disease. Since an average of 2–4% of European Caucasians are carriers of the PiZ allele, this fact should be considered during genetic counseling of patients [44].

*GJB2*. As a result of our study, three different heterozygous *GJB2* variants were identified: p.M34T (rs35887622), p.V37I (rs72474224), and c.-23+1G>A (rs80338940). The most common variant was p.M34T, for which HF was 5.71% (84.7% among all detected *GJB2* variants). In two US screenings for carriage of the p.M34T variant, AF was 1.56% and 0.5%, respectively [45,46]. In the Latvian population, the HF of p.M34T was 3.1% [47], and in the UK the AF was 1.984% [48]. This variant is a non-inactivating missense gene mutation, which leads to the replacement of the amino acid methionine with threonine, resulting in less pronounced hearing impairment and with a relatively late onset and progression [48]. Two other variants identified in this study, p.V37I and c.-23+1G>A, are rarer, and the HF was 0.8 and 0.24%, respectively. Variant p.V37I is most common in Taiwan (HF is up to 11.6%) [49].

In the Russian patients, the frequency of c.-23+1G>A was 3.4% [50], and in a large group of hearing-impaired patients (N = 580) living in the subarctic region of Russia (Sakha Republic) the most common pathogenic *GJB2* variant were c.-23+1G>A (51.82%) and c.109G>A (2.37%) [51].

The variant c.35delG (rs80338939) is rather common among Russian patients with SNHL. In one study the frequency was 79.6% [50], and in another, it was 23.5% [52]. The HF among Russian population in the Ekaterinburg region was 2.2% [53]. In our study, we did not find any carrier of this variant. One of the reasons could be the incorrect work of the assay (ANEPWEH) may be due to the absence of heterozygous samples in the study as positive controls.

The evaluation of AF of the *CFTR*, *PAH*, *SERPINA1*, and *GJB2* variants in the Russian population contributed to estimate the potential number of detected carrier couples by the custom panel. Comparing the data on the frequency of patients with CF, PKU, SNHL, and A1ATD in Russia [8,12,16,21] and the results of our study, we can conclude that the developed diagnostic panel allows for predicting the risk of the birth of offspring with PKU or SNHL in most cases, the risk of birth of a child with A1ATD in more than 60%, and an offspring with CF in about half of the cases. With regard to CF, only 7 of the 66 variants included in the panel were identified in the study population. Probably, undetected variants are rarer and the sample size was insufficient to detect them. Thus, we can suspect that our panel is able to detect more than only half of CF carriers. Unfortunately, we were not able to take into account penetrance of the genes variants when assessing disease burden, which is one of our study limitations.

In order to develop this diagnostic panel for carrier screening, a limited number of variants associated with CF, PKU, A1ATD, and SNHL in Russia were included. However, the panel has identified the vast majority of carriers of these recessive diseases in the population. Further, this method is easier for clinical interpretation compared to the next generation sequencing, as the pathogenicity of these variants is known. On the basis of that mentioned above and the availability of applied technology, we believe this panel is an effective approach to carrier screening for common recessive diseases.

## 5. Conclusions

A custom panel on the basis of a useful, relatively fast, cheap and reliable tool for application in molecular diagnostic and screening was developed to identify heterozygous carriage of *CFTR*, *PAH*, *SERPINA1*, and *GJB2* variants among Caucasians in the Russian population. The HF in the Russian population was 16.87% or 1:6 (CI95%: 14.76–19.00% by the Clopper–Pearson exact method): in *CFTR*—2.81% (1:36), *PAH*—2.33% (1:43), *SERPINA1*—4.90% (1:20), and *GJB2*—6.83% (1:15). The obtained data have shown that this panel allows us to detect couples with a high risk of having a child with CF, PKU, A1ATD, or SNHL. A high frequency of the detected variants in the Russian population makes carrier screening worthwhile.

## Figures and Tables

**Table 1:** Identified variants in the *CFTR, PAH, SERPINA1*, and *GJB2* genes among 1244 participants of the ESSE-Vologda study.

Gene	HGVS	dbSNP	Number of Identified Alleles	Variant Proportion among All Identified Variants, %	HF, %	At 95% CI, %	AF in the ESSE-Vologda Study, %	AF by [28]	AF (European (Non-Finnish), EXAC, GNOMAD Exome, GNOMAD Genome), % [29]
***CFTR***	p.F508del	rs113993960	21	60	1.69	1.05–2.57	0.84		1.06
	p.L138dup	rs397508686	4	11.43	0.32	0.09–0.82	0.16		0 *
	CFTRdele2.3, c.54-5940_273+10250del21080; p.S18Rfs*16	hg19:: chr7:117138367-117159446	3	8.57	0.24	0.05–0.7	0.12		0.013 **
	p.L88Ifs*22	rs121908769	3	8.57	0.24	0.05–0.7	0.12		0.04
	p.R117H	rs78655421	2	5.71	0.16	0.02–0.58	0.08	0.1445	0.26
	p.G542*	rs113993959	1	2.86	0.08	0.00–0.45	0.04	0.07236	0.03
	c.3718-2477C>T	rs75039782	1	2.86	0.08	0.00–0.45	0.04		0 **
		Total	35		2.81	1.97–3.89	1.40		
***PAH***	p.R408W	rs5030858	19	65.52	1.53	0.92–2.37	0.76	0.7959	0.11
	p.A403V	rs5030857	3	10.34	0.24	0.05–0.7	0.12	0.07236	0.09
	p.R261Q	rs5030849	2	6.9	0.16	0.02–0.58	0.08		0.04
	p.I306V	rs62642934	2	6.9	0.16	0.02–0.58	0.08		0.0015
	p.L48S	rs5030841	1	3.44	0.08	0.00–0.45	0.04		0.01
	c.1066-11G>A	rs5030855	1	3.44	0.08	0.00–0.45	0.04		0.04
	c.1315+1G>A	rs5030861	1	3.44	0.08	0.00–0.45	0.04		0.06
		Total	29		2.33	1.57–3.33	1.17		
***SERPINA1***	p.E288V	rs17580	33	55	2.65	1.83–3.71	1.33	1.2	3.04
	p.E366K	rs28929474	26	43.33	2.09	1.37–3.05	1.05	0.8671	1.83
	p.D280V	rs121912714 ^#^	1	1.67	0.16 ^#^	0.00–0.86 ^#^	0.08 ^#^	0.07215	0.07
		Total	60		4.90	3.7–6.17	2.45		
***GJB2***	p.M34T	rs35887622	72	84.7	5.71 ^§^	4.48–7.14	2.89	1.4	1.22
	p.V37I	rs72474224	10	11.76	0.80	0.39–1.47	0.40	0.5072	0.19
	c.-23+1G>A	rs80338940	3	3.53	0.24	0.05–0.7	0.12		0.032 **
		Total	85		6.83	5.42–8.29	3.42		
**Total**			209		16.87	14.76–19.00	8.44		

AF—allele frequency. HF—frequency of heterozygotes. ^#^ estimated on 642 samples; ^§^ frequency of homo- and heterozygotes. * GNOMAD Exome. ** GNOMAD Genome.

## Supplementary Materials

The following are available online at https://www.mdpi.com/2075-4426/10/3/140/s1, Table S1: The variants of the *CFTR*, *PAH*, *SERPINA1*, and *GJB2* genes included in the custom panel.
