# Supplementary material for: A Data-Driven Approach to Carrier Screening for Common Recessive Diseases
Source: J Pers Med. 2020 Sep 22;10(3):140. doi: 10.3390/jpm10030140 (PMC7563953; doi:10.3390/jpm10030140)
Supplement: Supplementary file 1 [file jpm-10-00140-s001.pdf]

Table S1. The variants of the *CFTR*, *PAH*, *SERPINA1*, and *GJB2* genes included in the custom panel.

dbSNP	Gene	HGVS	Assay ID	Allele Frequency (Total) ExAC; *GNOMAD Exome; **GNOMAD Genome [29]	Allele Frequency European (Non-Finnish) ExAC; *GNOMAD Exome; **GNOMAD Genome [29]
rs121908793	*CFTR*	NM_000492.3: c.580-1G>T	C_151693825_10	0.00000824	0.000015
rs77932196	*CFTR*	NM_000492.3: c.1040G>C; NP_000483.3: p.R347P	C___656878C_30	0.00000824	0.000015
rs121909012	*CFTR*	NM_000492.3: c.2551C>T; NP_000483.3: p.R851*	C____656779_20	0.00000825	0
rs121908805	*CFTR*	NM_000492.3: c.1397C>A; NP_000483.3: p.S466*	C_64676233C_10	0.00000826	0.000015
rs121908805	*CFTR*	NM_000492.3: c.1397C>G; NP_000483.3: p.S466*	C_64676233D_20	0.00000826	0.000015
rs121908761	*CFTR*	NM_000492.3: c.3276C>A; NP_000483.3: p.Y1092*	C_26083714C_10	0.00000828	0.0000151
rs387906378	*CFTR*	NM_000492.3: c.3532_3535dupTCAA; NP_000483.3: p.(Thr1179Ilefs*17)	ANFVPYF	0.00000828	0.000015
rs121908812	*CFTR*	NM_000492.3: c.2012delT; NP_000483.3: p.(Leu671*)	C_151693914_30	0.00000833	0.0000152
rs78194216	*CFTR*	NM_000492.3: c.3196C>T; NP_000483.3: p.R1066C	C__26083724_20	0.00000837	0.0000152
rs121908770	*CFTR*	NM_000492.3: c.442delA; NP_000483.3: p.(Ile148Leufs*5)	C_151693817_20	0.00000847	0.0000154
rs121908751	*CFTR*	NM_000492.3: c.274G>A; NP_000483.3: p.E92K	C_32545261C_10	0.00000855	0.0000155
rs121908751	*CFTR*	NM_000492.3: c.274G>T; NP_000483.3: p.E92*	C_32545261D_20	0.00000855	0.0000155
rs76151804	*CFTR*	NM_000492.3: c.3140-26A>G	C_100964965_10	0.0000141	0.0000273
rs121908763	*CFTR*	NM_000492.3: c.3587C>G; NP_000483.3: p.S1196*	C__32295560_20	0.0000165	0.0000301
rs77932196	*CFTR*	NM_000492.3: c.1040G>A; NP_000483.3: p.R347H	C___656878D_20	0.0000165	0.000015
rs121908746	*CFTR*	NM_000492.3:c.2052dup; NP_000483.3:p.Gln685fs	C_172767550_10	0.0000167	0.0000152
rs121908748	*CFTR*	NM_000492.3: c.1766+1G>A	C_151693906_10	0.0000167	0.0000303
rs121908760	*CFTR*	NM_000492.3: c.2125C>T; NP_000483.3: p.R709*	C__32295645_30	0.0000167	0

rs397508464	*CFTR*	NM_000492.3: c.293A>G; NP_000483.3: p.Q98R	C_201397567_10	0.0000167	0
rs121908749	*CFTR*	NM_000492.3: c.223C>T; NP_000483.3: p.R75*	C__64676210_20	0.0000247	0.000015
rs113993958	*CFTR*	NM_000492.3: c.328G>C; NP_000483.3: p.D110H	C____656922_20	0.0000249	0.0000452
rs77035409	*CFTR*	NM_000492.3: c.3691delT; NP_000483.3: p.(Ser1231Profs*4)	C_151693965_20	0.0000249	0
rs79850223	*CFTR*	NM_000492.3: c.3472C>T; NP_000483.3: p.R1158*	C__32545051_10	0.0000416	0.0000454
rs121908745	*CFTR*	NM_000492.3: c.1519_1521delATC; NP_000483.3: p.(Ile507del)	C_151693868_10	0.0000495	0.00003
rs397508200	*CFTR*	NM_000492.3: c.1393-1G>A	C_201387583_10	0.0000495	0
rs74767530	*CFTR*	NM_000492.3: c.3484C>T; NP_000483.3: p.R1162*	C__64676246_10	0.0000498	0.0000604
rs121908746	*CFTR*	NM_000492.3: c.2052delA; NP_000483.3: p.(Lys684Asnfs*38)	C_172767547_20	0.0000585	0.0000912
rs121909019	*CFTR*	NM_000492.3: c.3197G>A; NP_000483.3: p.R1066H	C__26083723_20	0.0000585	0.0000759
rs78756941	*CFTR*	NM_000492.3: c.489+1G>T	C_100964806_20	0.0000619	0.0000973
rs121909011	*CFTR*	NM_000492.3: c.1000C>T; NP_000483.3: p.R334W	C____656883_30	0.0000659	0.000045
rs75961395	*CFTR*	NM_000492.3: c.254G>A; NP_000483.3: p.G85E	C__32545286_20	0.000066	0.0001
rs80224560	*CFTR*	NM_000492.3: c.2657+5G>A	C_100964951_10	0.0000824	0.0001
rs76713772	*CFTR*	NM_000492.3: c.1585-1G>A	C_100964916_20	0.0000913	0.0002
rs397508276	*CFTR*	NM_000492.3: c.1705T>G; NP_000483.3: p.Y569D	C_201390110_10	0.0000994	0
rs121908755	*CFTR*	NM_000492.3: c.1646G>A; NP_000483.3: p.S549N	C__32545131_20	0.0001	0.0000301
rs121908811	*CFTR*	NM_000492.3: c.3659delC; NP_000483.3: p.(Thr1220Lysfs*8)	C_172767557_20	0.0001	0.0000752
rs145449046	*CFTR*	NM_000492.3: c.2374C>T; NP_000483.3: p.R792*	C__26083745_30	0.0001	0.0000157
rs74597325	*CFTR*	NM_000492.3: c.1657C>T; NP_000483.3: p.R553*	C__27861430_20	0.0001	0.0002
rs75527207	*CFTR*	NM_000492.3: c.1652G>A; NP_000483.3: p.G551D	C__32545127_20	0.0001	0.0003
rs80034486	*CFTR*	NM_000492.3: c.3909C>G; NP_000483.3: p.N1303K	C__32544994_20	0.0001	0.0002

rs113993959	*CFTR*	NM_000492.3: c.1624G>T; NP_000483.3: p.G542*	C__11399026_30	0.0003	0.0004
rs121908747	*CFTR*	NM_000492.3: c.3528delC; NP_000483.3: p.(Lys1177Serfs*15)	C_151693962_30	0.0003	0.0006
rs121908769	*CFTR*	NM_000492.3: c.262_263delTT; NP_000483.3: p.(Leu88Ilefs*22)	C_151693788_20	0.0003	0.0004
rs75541969	*CFTR*	NM_000492.3: c.3454G>C; NP_000483.3: p.D1152H	C__32545067_20	0.0003	0.0004
rs77834169	*CFTR*	NM_000492.3: c.349C>T; NP_000483.3: p.R117C	C__27540773_10	0.0003	0.0006
rs77010898	*CFTR*	NM_000492.3: c.3846G>A; NP_000483.3: p.W1282*	C__32545014_20	0.0004	0.0007
rs78655421	*CFTR*	NM_000492.3: c.350G>A; NP_000483.3: p.R117H	C__26083773_20	0.0015	0.0026
rs113993960	*CFTR*	NM_000492.3: c.1521_1523delCTT; NP_000483.3: p.(Phe508del)	C_151693869_10	0.0068	0.0106
rs397508686	*CFTR*	NM_000492.3: c.413_415dupTAC; NP_000483.3: p.(Leu138dup)	ANKA7N7	0.000003988*	0*
hg19:: chr7:117138367 -117159446	*CFTR*	NM_000492.3: c.54-5940_273+10250del21080; NP_000483.3: p.(Ser18Argfs*16)	C990000002A_10	0.00004610**	0.0001312**
hg19:: chr7:117138367 -117159446	*CFTR*	NM_000492.3: c.54-5940_273+10250del21080; NP_000483.3: p.(Ser18Argfs*16)	C990000002B_20	0.00004610**	0.0001312**
rs121908799	*CFTR*	NM_000492.3: c.2051_2052delAAinsG; NP_000483.3: p.(Lys684Serfs*38)	C172767555C_30	0.004013*	0.00000891 *
rs121908803	*CFTR*	NM_000492.3: c.613C>T; NP_000483.3: p.P205S	C__32545236_30	0.000003977 *	0 *
rs397508158	*CFTR*	NM_000492.3: c.1116+1G>A	C_201385092_10	0.000003991 *	0.00000884 *
rs397508573	*CFTR*	NM_000492.3: c.3476C>T; NP_000483.3: p.S1159F	C_400040945_10	0.000003991*	0.000008837*
rs75039782	*CFTR*	NM_000492.3: c.3718-2477C>T	C_100965026_20	0.00006376**	0 **
rs121908776	*CFTR*	NM_000492.3: c.1545_1546delTA; NP_000483.3: p.(Tyr515*)	C_151693870_20	NA	NA
rs121908771	*CFTR*	NM_000492.3: c.531delT; NP_000483.3: p.(Ile177Metfs*12)	C_151693822_20	0.000007978 *	0.00001764 *
rs121908778	*CFTR*	NM_000492.3: c.1911delG; NP_000483.3: p.(Gln637Hisfs*26)	C_151693912_20	NA	NA
rs397508152	*CFTR*	NM_000492.3: c.1075_1079delCAAACinsAAAAA; NP_000483.3:	C_203006811_10	NA	NA

		p.(Gln359_Thr360delinsLysLys)			
rs397508184	*CFTR*	NM_000492.3: c.1243_1247delAACAA; NP_000483.3: p.(Asn415*)	C_400015123_10	NA	NA
rs397508449	*CFTR*	NM_000492.3: c.287C>A; NP_000483.3: p.A96E	C_400029971_10	NA	NA
rs397508538	*CFTR*	NM_000492.3: c.3310G>T; NP_000483.3: p.E1104*	C_201400019_10	NA	NA
rs397508612	*CFTR*	NM_000492.3: c.3816_3817delGT; NP_000483.3: p.(Ser1273Leufs*28)	C_400040984_10	NA	NA
rs397508616	*CFTR*	NM_000492.3: c.3844T>C; NP_000483.3: p.W1282R	C_400040988_10	NA	NA
rs397508706	*CFTR*	NM_000492.3: c.4251delA; NP_000483.3: p.(Glu1418Argfs*14)	C_201404927_10	NA	NA
rs104894413	*GJB2*	NM_004004.5: c.131G>C; NP_003995.2: p.W44S	C__34696726_10	0.00000825	0
rs28931592	*GJB2*	NM_004004.5: c.476A>T; NP_003995.2: p.D159V	C__32403574_10	0.0000165	0.00003
rs80338950	*GJB2*	NM_004004.5: c.551G>C; NP_003995.2: p.R184P	C__11406734_10	0.0000413	0.0000451
rs1801002	*GJB2*	NM_004004.5: c.35G>T; NP_003995.2: p.G12V	C___7552294_10	0.0000501	0
rs80338947	*GJB2*	NM_004004.5: c.358_360delGAG; NP_003995.2: p.(Glu120del)	C_154794101_20	0.0000661	0.0001
rs111033253	*GJB2*	NM_004004.5: c.313_326delAAGTTCATCAAGGG; NP_003995.2: p.(Lys105Glyfs*5)	C_154794103_10	0.0000991	0.0001
rs80338944	*GJB2*	NM_004004.5: c.231G>A; NP_003995.2: p.W77*	C__27536852_10	0.0001	0.00003
rs80338948	*GJB2*	NM_004004.5: c.427C>T; NP_003995.2: p.R143W	C__11406749_10	0.0002	0.0000901
rs80338943	*GJB2*	NM_004004.5: c.235delC; NP_003995.2: p.(Leu79Cysfs*3)	C_154794105_20	0.0004	0.000015
rs80338942	*GJB2*	NM_004004.5: c.167delT; NP_003995.2: p.(Leu56Argfs*26)	C_154794111_20	0.0007	0.0011
rs80338939	*GJB2*	NM_004004.5: c.35delG; NP_003995.2: p.(Gly12Valfs*2)	ANEPWEH	0.006	0.0088
rs72474224	*GJB2*	NM_004004.5: c.109G>A; NP_003995.2: p.V37I	C__11406800_20	0.0066	0.0019
rs35887622	*GJB2*	NM_004004.5: c.101T>C;	C__11406801_10	0.0085	0.0122

		NP_003995.2: p.M34T			
rs28931593	*GJB2*	NM_004004.5: c.224G>A; NP_003995.2: p.R75Q	C__29586514_10	0.000006978*	0*
rs80338940	*GJB2*	NM_004004.5: c.-23+1G>A	C_154794206_10	0.000191755**	0.000324886**
rs104894401	*GJB2*	NM_004004.5: c.428G>A; NP_003995.2: p.R143Q	C__11406748_10	NA	NA
rs104894402	*GJB2*	NM_004004.5: c.223C>T; NP_003995.2: p.R75W	C__11406780_10	NA	NA
rs5030850	*PAH*	NM_000277.2: c.781C>T; NP_000268.1: p.R261*	C__27528517_10	0.00000824	0.000015
rs62514936	*PAH*	NM_000277.2: c.664_665delGA; NP_000268.1: p.(Asp222*)	C_104519192_20	0.00000824	0.000015
rs62642906	*PAH*	NM_000277.2: c.47_48delCT; NP_000268.1: p.(Ser16*)	C__89759003_20	0.00000824	0.000015
rs62642934	*PAH*	NM_000277.2: c.916A>G; NP_000268.1: p.I306V	C__27863391_20	0.00000825	0.000015
rs62508646	*PAH*	NM_000277.2: c.1045T>C; NP_000268.1: p.S349P	ANCE79N	0.0000333	0.0000453
rs62507288	*PAH*	NM_000277.2: c.168+5G>C	ANAAENR	0.0000412	0.000045
rs62507321	*PAH*	NM_000277.2: c.168+5G>T	C__89758964_10	0.0000412	0.000045
rs62508698	*PAH*	NM_000277.2: c.838G>A; NP_000268.1: p.E280K	ANDJ2UK	0.0000412	0.000045
rs76296470	*PAH*	NM_000277.2: c.331C>T; NP_000268.1: p.R111*	C__33221788_10	0.0000494	0.00003
rs5030846	*PAH*	NM_000277.2: c.727C>T; NP_000268.1: p.R243*	C__27863366_10	0.0000495	0.0000901
rs5030847	*PAH*	NM_000277.2: c.754C>T; NP_000268.1: p.R252W	C____657294_10	0.0000495	0.00009
rs62508588	*PAH*	NM_000277.2: c.728G>A; NP_000268.1: p.R243Q	C__64676825_10	0.0000743	0.00003
rs5030841	*PAH*	NM_000277.2: c.143T>C; NP_000268.1: p.L48S	C__61900229_20	0.0000824	0.0001
rs5030851	*PAH*	NM_000277.2: c.842C>T; NP_000268.1: p.P281L	C__27863379_10	0.0000988	0.0002
rs5030843	*PAH*	NM_000277.2: c.473G>A; NP_000268.1: p.R158Q	C__32330008_10	0.000099	0.0002
rs5030849	*PAH*	NM_000277.2: c.782G>A; NP_000268.1: p.R261Q	AN9HNAZ	0.0003	0.0004
rs5030855	*PAH*	NM_000277.2: c.1066-11G>A	C__32330003_20	0.0003	0.0004
rs5030861	*PAH*	NM_000277.2: c.1315+1G>A	C__32329996_10	0.0003	0.0006

rs5030853	*PAH*	NM_000277.2: c.898G>T; NP_000268.1: p.A300S	C__27863387_20	0.0004	0.0007
rs5030857	*PAH*	NM_000277.2: c.1208C>T; NP_000268.1: p.A403V	C__27863418_20	0.0005	0.0009
rs5030860	*PAH*	NM_000277.2: c.1241A>G; NP_000268.1: p.Y414C	ANGZJJD	0.0005	0.0008
rs5030858	*PAH*	NM_000277.2: c.1222C>T; NP_000268.1: p.R408W	C__32329998_10	0.0007	0.0011
rs199475649	*PAH*	NC_000012.11:g.103260374_103260441del	ANH6D4A	NA	NA
rs1802959	*SERPINA1*	NM_000295.4: c.1078G>A; NP_000286.3: p.A360T	AN7DUP3	0.00000838	0.0000152
rs199422210	*SERPINA1*	NM_000295.4: c.552C>G; NP_000286.3: p.Y184*	C__11665819_20	0.0000165	0.00003
rs199422209	*SERPINA1*	NM_000295.4: c.1178C>T; NP_000286.3: p.P393L	C_190117974_10	0.000033	0.0000599
rs28931569	*SERPINA1*	NM_000295.4: c.194T>C; NP_000286.3: p.L65P	C____594704_10	0.0000412	0.000075
rs11558261	*SERPINA1*	NM_000295.4: c.415G>A; NP_000286.3: p.G139S	C____594710_20	0.0000989	0
rs55819880	*SERPINA1*	NM_000295.4: c.230C>T; NP_000286.3: p.S77F	C____594705_10	0.0002	0.0001
rs121912714	*SERPINA1*	NM_000295.4: c.839A>T; NP_000286.3: p.D280V	C__64676988_10	0.0004	0.0007
rs28931570	*SERPINA1*	NM_000295.4: c.187C>T; NP_000286.3: p.R63C	C____594703_10	0.001	0.0016
rs28929474	*SERPINA1*	NM_000295.4: c.1096G>A; NP_000286.3: p.E366K	C__34508510_10	0.0117	0.0183
rs17580	*SERPINA1*	NM_000295.4: c.863A>T; NP_000286.3: p.E288V	C____594695_20	0.0201	0.0304
